# Susceptibility of 316L Stainless Steel Structures to Corrosion Degradation in Salivary Solutions in the Presence of Lactic Acid

**DOI:** 10.3390/jfb14110535

**Published:** 2023-10-30

**Authors:** Lidia Benea, Iulian Bounegru, Elena Roxana Axente, Daniela Buruiană

**Affiliations:** 1 Competences Centre: Interfaces-Tribocorrosion-Electrochemical Systems (CC-ITES), Dunărea de Jos University of Galați, 47 Domnească Street, 00008 Galati, Romania; daniela.buruiana@ugal.ro; 2 Faculty of Medicine and Pharmacy, Dunărea de Jos University of Galați, 35 Al. I. Cuza Street, 800010 Galati, Romania; elena.axente@ugal.ro; 3 Faculty of Engineering, Dunărea de Jos University of Galați, 47 Domnească Street, 800008 Galati, Romania

**Keywords:** 316L stainless steel, surface reactivity, corrosion susceptibility, saliva solution, lactic acid, specific resistance, corrosion current density

## Abstract

In the field of healthcare and dentistry, 316L stainless steel is widely used for its corrosion resistance. However, the presence of lactic acid in salivary solutions can affect its surface reactivity. This study employed electrochemical methods to investigate the influence of lactic acid on 316L stainless steel’s corrosion resistance in Fusayama Meyer saliva and saliva doped with varying lactic acid concentrations. The results revealed a significant decrease in polarization resistance as the lactic acid concentration increased, despite a shift toward more positive corrosion potentials. Consequently, the study suggests that the lactic acid presence in salivary solutions should be considered when evaluating the corrosion susceptibility of 316L stainless steel devices.

## 1. Introduction

Corrosion, the destructive process resulting from a material’s chemical or electrochemical interactions with its surrounding environment, can severely compromise the integrity of materials used in the medical field, obstructing treatment steps by deteriorating metals within the environment [1,2,3]. Recognizing the pervasive and detrimental effects of corrosion, scientific efforts have been directed toward fabricating materials resistant to such decay or materials that retard this destructive process over an extended timeframe [3,4].

Stainless steel, an iron alloy renowned for its high strength, corrosion resistance, ability to withstand sterilization temperatures, and capacity to be manufactured into complex geometries, including through 3D printing, has found extensive application in a multitude of sectors [5,6]. Within healthcare, it has emerged as an ideal material for various types of equipment, ranging from prosthetic devices, bone fixation tools, artificial heart components, and prostheses [7,8]. The versatility of stainless steel extends to dentistry, where it is utilized for equipment, pediatric dental crowns, orthodontic bracket materials, and arches [9,10,11].

The increasing prevalence of 316L stainless steel in dentistry can be attributed to its biocompatibility, as it poses no risk of infectious diseases or inflammatory responses within the human body [12,13]. The absence of toxic, immunogenic, and carcinogenic implications prioritizes tissue preservation, underscoring its suitability for dental applications [11,12,14].

A pivotal actor in the realm of biochemical reactions is lactic acid, a metabolite of pyruvic acid. It is generated during the reduction of pyruvic acid by the lactate dehydrogenase enzyme under anaerobic glucose metabolism [15]. Various human tissues, including skeletal muscle, resort to this metabolic pathway for energy production in oxygen-deprived conditions [15,16]. Lactic acid is also synthesized by lactic acid bacteria, significantly influencing the fermentation processes of numerous food and beverage items [15].

The food industry extensively utilizes lactic acid as an additive and preservative, while its broad range of industrial applications includes biodegradable polymer production and as a solvent in cosmetics and cleaning products [17]. Its biodegradable and non-toxic properties have positioned lactic acid as an appealing alternative to conventional solvents, demonstrating its potential for diverse industrial applications [6,17].

Within the oral cavity, the metabolic activities of oral bacteria, including Streptococcus mutants, Lactobacillus, and Actinomyces, lead to lactic acid production [15,16]. The oral environment’s pH, typically between 6.2 to 7.6, can decrease significantly, leading to enamel demineralization and dental caries, because of dietary sugar or fermentable carbohydrate consumption [15,16,18].

This study’s core objective is to explore the corrosion behavior of 316L stainless steel, a popular material in dental and orthodontic applications, when exposed to varying concentrations of lactic acid. Lactic acid is a prevalent acid naturally found in the oral cavity, which significantly contributes to the degradation of metallic dental materials.

A key aspect of this investigation is the formation of a protective chromium oxide (Cr_2_O_3_) passive film on the surface of 316L stainless steel. This film serves as a corrosion barrier, yet the presence of lactic acid in saliva may impact its stability and integrity.

Assessing the influence of lactic acid concentration on 316L stainless steel’s corrosion potential within the oral environment is crucial for understanding the material’s longevity and suitability for dental applications. Through unraveling the underlying corrosion mechanisms and trends, this study aims to impart valuable insights in order to catalyze the development of more corrosion-resistant dental materials, thereby facilitating improved clinical outcomes.

This study uniquely explores the corrosion behavior of 316L stainless steel in environments with varying lactic acid concentrations, mimicking the oral cavity’s conditions post consumption of acidic foods and beverages. Unlike previous research, our investigation focuses on the impact of lactic acid on the stability and integrity of the protective chromium oxide (Cr_2_O_3_) passive film on 316L stainless steel. Understanding the interaction between lactic acid and this corrosion-resistant film is crucial, as it provides valuable insights into the material’s longevity and performance in the dynamic oral environment.

Through a meticulous examination of the underlying corrosion mechanisms and trends in lactic acid environments, this study aims to fill the knowledge gap in the literature, offering valuable insights for developing more corrosion-resistant dental materials. Ultimately, our findings are expected to facilitate improved clinical outcomes and contribute to a broader understanding of 316L stainless steel’s suitability for dental applications in various acidic environments.

## 2. Materials and Methods

The 316L stainless steel material is used for many biomedical applications like prosthetic devices, bone fixation equipment, artificial stents for the heart, and orthodontic bracket devices; therefore, it is important to have a better understanding of the material’s corrosion behavior in different body fluids and extreme conditions, like the presence of lactic acid. This study uses 316L stainless steel with the chemical composition as shown in Table 1.

The accurate determination of hydrogen content in steel samples requires thorough preparation and cleaning. Different methods exist depending on the specific requirements and conditions [19]. In our study, we used a cleaning method recommended by Arroyo et al. to ensure reliable and consistent results in determining hydrogen content in 316L stainless steel.

The HCl treatment effectively removed the passive oxide layer found on stainless steel surfaces. This provided an oxide-free surface at the beginning of the experiment, which served as a consistent and uniform starting point for all samples. This ensured reliable and reproducible experimental results.

We found the HCl treatment beneficial for two reasons. First, it acted as an effective cleaning agent, removing any surface contaminants, residues, or films that may have been present. This ensured that the surface was clean and free of impurities before the start of the experiment. Second, it aided in the removal of passive oxide layers, which naturally form on stainless steel surfaces. While these oxide layers are often protective, they can influence the corrosion resistance and behavior of the metal [19].

The 316L stainless steel samples used for electrochemical tests were cut into pieces measuring 2.5 cm · 2.5 cm · 2 mm. We soldered them using copper wire to establish electrical contact and embedded them in epoxy resin to isolate all surfaces and the copper wire contacts. This resulted in a well-defined and measurable active surface area of 3.2 cm^2^. Before each experiment, the samples were cleaned using a 0.5 M solution of sodium hydroxide (NaOH), washed, treated with 10% hydrochloric acid (HCl), washed again with water, and, finally, rinsed with distilled water.

For the preparation of test solutions, reagents for analysis (p.a.) from Sigma Aldrich chemicals were used. The basic electrolyte used for the in vitro investigation of the resistance of 316L stainless steel is the biological solution that simulates saliva, Saliva Fusayama Meyer (SFM), whose composition is given in Table 2.

To simulate the metabolic product from the oral cavity or produced by the muscles during physical effort, lactic acid (LA) was added to this biological solution in different concentrations. Lactic acid used in this research work is produced by Sigma Aldrich, located in St. Louis, Missouri, USA, at 85% p.a.

With the aid of Multiparameter Sension+, the important parameters of each solution were measured as the pH, the conductivity, and the salinity, as shown in Table 3. This experiment was carried out at a temperature of 37 °C ± 0.5 to simulate the real condition of the human body.

To evaluate and monitor the corrosion resistance of 316L stainless steel in the working solutions specified in Table 3, an electrochemical cell with three electrodes connected to an electrochemical workstation is used with a constant 150 mL solution volume inside the electrochemical cell.

The working electrode (WE) consists of 316L stainless steel prepared as specified. The counter electrode (CE) is a platinum mesh with an active surface of 3 cm^2^, while the reference electrode (RE) is Ag/AgCl in saturated KCl solution, with a constant potential of +199 mV vs. the standard hydrogen electrode (NHE). All experiments were carried out and repeated three times to check reproducibility. All experiments were carried out at 37 ± 2 °C and repeated three times to check reproducibility.

**Figure 1:**
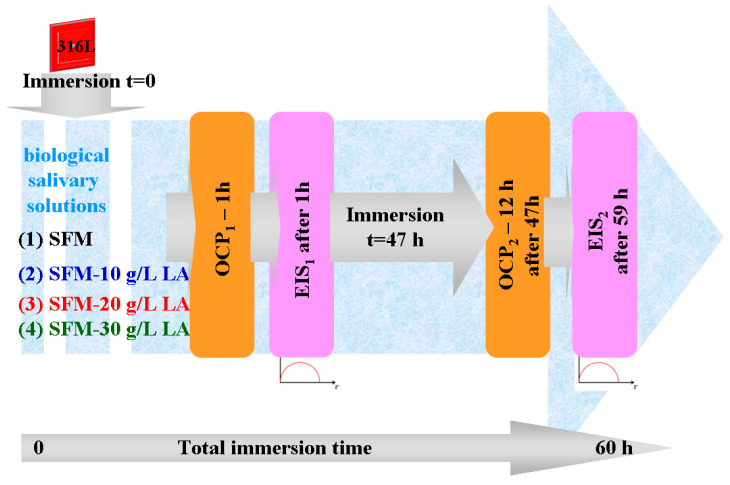
Schematic representation of the protocol applied to evaluate the corrosion resistance of 316L stainless steel in SFM and SFM doped with LA in different concentrations.

For each working solution, after immersing the sample in the electrochemical cell and connection to the electrochemical workstation, it is applied an electrochemical protocol of measurements is shown in Figure 1 and consists of the following steps:

We monitor the open circuit potential (OCP_1_) for one hour until we are sure that it has arrived at a steady state of stainless-steel surface in the tested solution.

OCP measurements during the first hour of immersion were conducted to monitor the initial stabilization of the open circuit potential of 316L stainless steel in various working solutions. This short duration was chosen to observe the immediate response and initial stabilization of the material in the solutions, which was crucial for the study’s objectives. Additionally, OCP measurements were conducted for 12 h after 47 h of immersion (referred to as OCP2) to evaluate the long-term surface stability of the stainless steel in the working solutions.

We monitor the electrochemical impedance spectroscopy (EIS_1_) at free potential with an amplitude of sinusoidal potential of 10 mA, from a high frequency of 100 kHz to a low frequency of 1 mHz with 10 frequencies per decade.

We leave the 316L samples immersed in the studied solutions for 47 h and repeat the cycle of open circuit potential and electrochemical impedance spectroscopy measurements as follows:

We measure the open circuit potential (OCP_2_) for 12 h to evaluate the surface stability after a long immersion time (47 h).

We monitor the electrochemical impedance spectroscopy (EIS_2_) at free potential with an amplitude of sinusoidal potential of 10 mA, from a high frequency of 100 kHz to a low frequency of 1 mHz with 10 frequencies per decade. This measurement gives us information about any changes and reactivity occurring on the 316L surface after 59 h in the studied solutions.

Separately, on other samples, the linear polarization diagrams were performed on 316L stainless steel in each solution to measure the corrosion current density and compare the effect of lactic acid. Thus, the linear polarization curves were measured in the potential range of ±200 mV around the free potential with a scan rate of 1 mV·s^−1^.

The surface morphology of pure 316L stainless steel was examined before and after a 5-h immersion in the working solutions using an Optika IM5 optical microscope.

To maintain the integrity of our results, all experimental conditions, such as temperature (maintained at 37 °C) and immersion duration, were kept consistent across varying lactic acid concentrations.

## 3. Results and Discussion

### 3.1. Monitoring of OCP during the First Hour of Immersion (OCP_1_)

Before commencing with the OCP measurements, each 316L stainless steel sample underwent a standardized cleaning procedure. This ensured a uniform starting point for all experiments, irrespective of the solution concentration.

**Figure 2:**
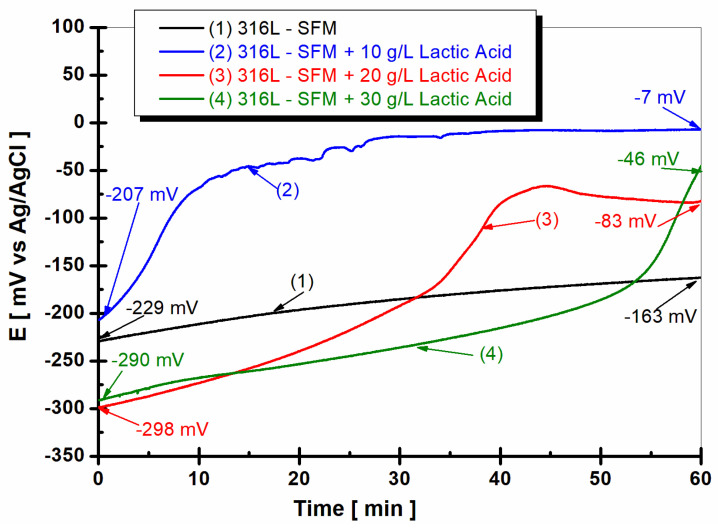
The evolution of the open circuit potential of 316L stainless steel during the first hour after immersion in (1) SFM; (2) SFM doped with 10 gL^−1^ LA; (3) SFM doped with 20 gL^−1^ LA; and (4) SFM doped with 30 gL^−1^ LA.

The results of OCP monitoring in the first hour after the immersion of 316L stainless steel in all working solutions are shown in Figure 2.

From Figure 2, it is observed that the open circuit potential of 316L stainless steel immersed in SFM shows an approximately constant value, with a small variation during the first hour of measurement from −229 mV to −163 mV vs. Ag/AgCl, as seen in Figure 2, curve (1). 

Doping the Fusayama Meyer solution with 10 gL^−1^ LA causes the free potential of the stainless steel to move towards more positive values, having at the start of the immersion the value of −207 mV vs. Ag/AgCl and climbing a lot towards more positive values, reaching at the end of the first hour of immersion the value of −7 mV vs. Ag/AgCl.

The highest concentration of LA, 10 and 20 g/L, causes a further shift of the open circuit potential to more negative values, having at immersion the values of −298 mV vs. Ag/AgCl, as seen in Figure 2, curve (3), and −290 mV, respectively, as seen in Figure 2, curve (4).

It is well-known that on a 316L stainless steel surface a Cr_2_O_3_, passive film is formed [20] and is named chromium trioxide or chromium sesquioxide or chromium (III) oxide, or chromia. Even if it is insoluble in water, it dissolves in acid to give hydrated chromium ions [Cr(H_2_O)_6_]^3+^. It reacts with alkali to yield chromite ions.

Lactic acid, with the chemical formula C_3_H_6_O_3_ or CH_3_CHOHCOOH, or HC_3_H_5_O_3_, is a weak organic acid known to form complex combinations or chelates when it reacts with metals or oxides. Specifically, lactic acid tends to form chelates with metal ions such as chromium and nickel that are present in the passive film of 316L stainless steel [21].

The observed data suggest a direct correlation between the concentration of lactic acid and the corrosion behavior of 316L stainless steel. This correlation implies an increased propensity for corrosion initiation and a destabilization of the passive film, as evidenced by a shift towards a more positive open circuit potential [22]. When comparing lower to higher concentrations of lactic acid, it appears that, at lower concentrations, lactic acid may enhance the protective properties of the passive film and stabilize it against corrosion. However, at higher lactic acid concentrations, the corrosive nature of the acid can lead to the breakdown of the passive film and the generation of reactive species that promote corrosion [23].

As the 316L stainless steel sample is immersed, an initial passive oxide layer forms. The growth, stability, and characteristics of this layer are contingent on various factors, including the pH of the solution, ambient temperature, and the presence of other reactive species. Over time, this oxide layer undergoes evolutionary changes, which are pivotal in determining the corrosion resistance of the steel [23].

Lactic acid’s interaction with metals, especially in the context of 316L stainless steel, is governed by its ability to form chelates. The metal-binding constant of lactate, especially with chromium and nickel, suggests a strong affinity. Furthermore, the solubility of lactate metal complexes indicates that these complexes are likely soluble, leading to potential disruptions in the passive film [24].

While many weak organic acids form chelates with metals, lactic acid’s unique molecular structure and properties might influence its interaction with the passive film differently. Unlike some other acids, lactic acid’s metal complexes have been observed to be particularly soluble, which might account for its pronounced effect on 316L stainless steel [24].

There is a possibility that lactic acid, upon interaction, forms a weak or porous surface layer on the stainless steel. Such a layer, if formed, could have implications on the corrosion resistance of the steel. Studies on organic acids, like citric acid, have shown the formation of such layers on metal surfaces, and it is crucial to understand if lactic acid behaves similarly.

The hypotheses presented herein are based on the experimental data obtained in this study, as observed in Figure 2, and are supported by existing literature on the interaction between weak organic acids and metallic surfaces [25]. Previous studies have shown that weak organic acids like lactic acid can form chelates or complexes with metals, influencing the stability of the passive film in a concentration-dependent manner [26].

### 3.2. EIS of 316L ss after 1 h from Immersion (EIS_1_)

**Figure 3:**
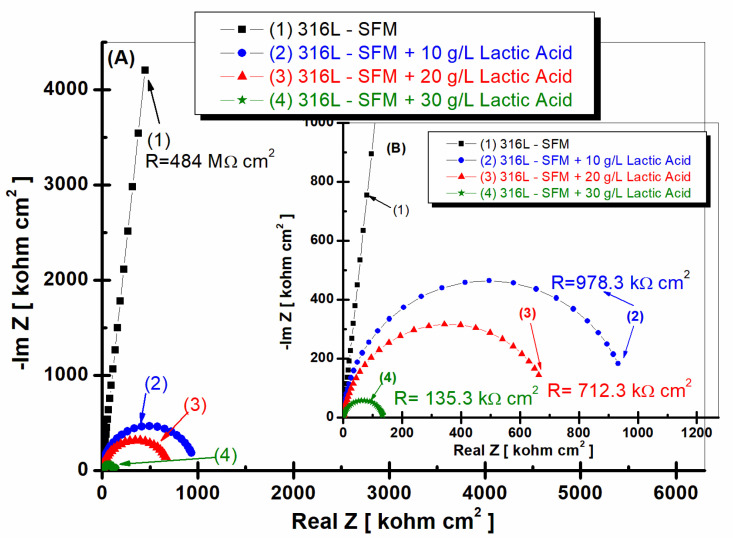
Nyquist plots of EIS results (filled symbols) and fitting with an equivalent electrical circuit (continuous line) of 316L stainless steel after 1 h of immersion in (1) SFM; (2) SFM doped with 10 g·L^−1^ LA; (3) SFM doped with 20 g·L^−1^ LA; and (4) SFM doped with 30 g·L^−1^ LA. Layer (**A**)—all frequency range; layer (**B**)—zoom to high frequency to better see smaller EIS.

**Figure 4:**
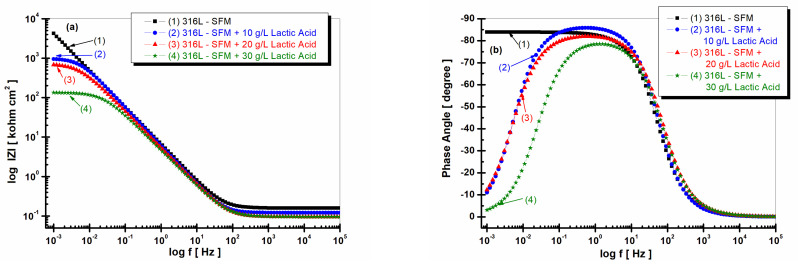
Bode plots of EIS results (filled symbols) and fitting with an equivalent electrical circuit (continuous line) of 316L stainless steel after 1 h of immersion in various solutions (1) SFM; (2) SFM doped with 10 g·L^−1^ LA; (3) SFM doped with 20 g·L^−1^ LA; and (4) SFM doped with 30 g·L^−1^ LA. (**a**) Impedance modulus vs. log frequency (log f [Hz]); (**b**) phase angle vs. log frequency (log f [Hz]). The horizontal axis represents the logarithm of frequency, ranging from 10^−3^ to 10^−5^ Hz.

Figure 3 and Figure 4 demonstrate the results of the EIS performed on 316L stainless steel after one hour of its immersion in SFM and saliva doped with different concentrations of LA, from 0 g·L^−1^ (saliva) to 10, 20, and 30 g·L^−1^.

The 316L stainless steel immersed only in SFM reveals a specific resistance value of 484 MΩ·cm^2^, suggesting an appreciable resistance to the flow of electrical current in this environment.

When the SFM is doped with 10 g·L^−1^ lactic acid, the resistance value of the 316L stainless steel notably dropped to 978.3 kΩ·cm^2^. This decrease in specific resistance signifies a decrease in the impedance experienced by the stainless steel in response to this specific lactic acid concentration.

Increasing the LA concentration to 20 g·L^−1^ of the SFM resulted in a further reduction in the specific resistance value to 712.3 kΩ·cm^2^. This lower resistance value points to a relatively higher electrical conductivity and stainless-steel surface reactivity as compared to the sample immersed only in Fusayama Meyer saliva.

The lowest specific resistance value in our study, 135.3 kΩ·cm^2^, is observed when the 316L stainless steel is immersed in SFM doped with 30 g·L^−1^ lactic acid. This suggests a decrease in the electrochemical impedance encountered by the stainless steel in the presence of a higher LA concentration.

Figure 4a,b shows the Bode plots of EIS results obtained after one hour from immersion time. The higher impedance modulus is revealed by 316L stainless steels when immersed in saliva solution, as seen in curve 1 from Figure 4a. The LA added to saliva solution in all studied concentrations causes a decrease in the impedance modulus with increasing lactic acid concentration, as seen in Figure 4a, curves 2, 3, and 4.

If 316L is immersed only in saliva, the phase angle shows a high constant value of 85 degrees over a wide frequency range, as seen in Figure 4b, curve (1); the presence of LA changes the phase angle curves, making them go through a maximum on a narrow range of frequencies to then decreasing to smaller values of the phase angle, as seen in Figure 4b, curves (2), (3), and (4). Thus, for the concentration of 10 g·L^−1^ of LA in the saliva, the value of the phase angle reaches its maximum at 85 degrees and drops rapidly to approximately 10 degrees, as seen in the curve (2) in Figure 4. At the concentration of 20 gL^−1^ LA in the saliva, the value of the phase angle reaches its maximum at approximately 80 degrees and also decreases at the value of 10 degrees, as seen in the curve (3) in Figure 4b. For the highest concentration of LA in the saliva, respectively, 30 g·L^−1^, the value of the phase angle reaches its maximum only at approximately 75 degrees, after which it decreases further below 5 degrees, as seen in the curve (4) in Figure 4b.

**Figure 5:**
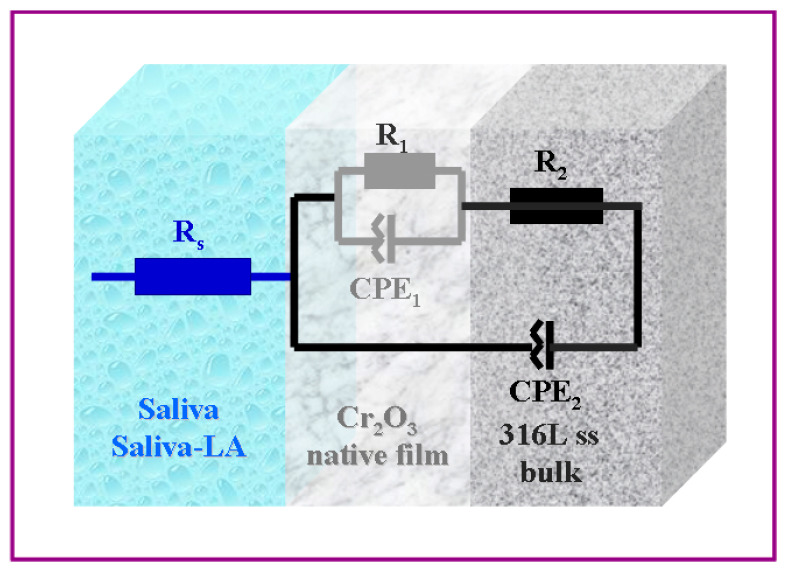
Randles equivalent electrical circuit fitting the interface of 316L stainless steel immersed in SFM and saliva doped with different concentrations of LA.

EIS, a non-destructive technique, delivers time-sensitive data not only about the inherent properties of materials but also about the progression of various processes, including corrosion degradation and other electrochemical activities. For the evaluation of the EIS results and the specific resistance or the polarization resistance, the experimental results of the EIS are fitted with an equivalent electrical circuit that best describes the interface of the 316L stainless steel through the natively formed Cr_2_O_3_ and the SFM solution. The fitting was carried out with Z_view_ software, and the accuracy of the results was verified by the chi-square value obtained at 10^−4^. The electrical equivalent circuit proposed is shown in Figure 5.

In Figure 5, the term R_s_ denotes the resistance of the solution. The pair [R_1_, CPE_1_] corresponds to the resistance and constant phase element that are ascribed to the passive film of Cr_2_O_3_. Conversely, the pair [R_2_, CPE_2_] signifies the resistance and constant phase element inherent to the bulk material of 316L stainless steel. 

Such an equivalent circuit of the 316L stainless steel immersed in saliva solution and saliva doped with lactic acid is described by the impedance of a non-ideal capacitor having a constant phase element instead of a double layer capacitance (CPE, *Q*), described by the equation [27,28,29,30]:

(1) $$Z_{CPE}=\frac{1}{Q{\left(j\omega \right)}^{\alpha }}$$

where α is the CPE exponent varied between 0 and 1, *ω* is an angular frequency (*ω* = 2πf), and *Q* measured in *F·s^−(1−α)^* or *Ω^−1^s^α^* is the CPE parameter. Interfacial capacitance is usually approximated by the value of *Q* when *α* → 1, but this approach is inaccurate [29]. Many theoretical and experimental works are carried out to understand the origin of CPE behavior, which is still controversial. In general, the behavior is attributed to the inhomogeneity of the studied surfaces, roughness, and porosity, but also to the adsorption of specific anions during the corrosion process degradation occurring on these surfaces [30,31].

The variations in R values across different lactate concentrations suggest alterations in the properties of the surface layer. As the concentration of lactate changes, it potentially influences the electrochemical interactions at the steel interface, leading to these observed variations. Over extended periods, it is hypothesized that the R values might trend toward stabilization across different lactate concentrations. This could indicate the eventual formation of a stable end-stage at the surface, irrespective of the initial lactate concentration. Such behavior would be indicative of the adaptive nature of the stainless-steel surface in response to varying electrochemical environments.

### 3.3. OCP Evolution after 47 h from Immersion (OCP_2_)

**Figure 6:**
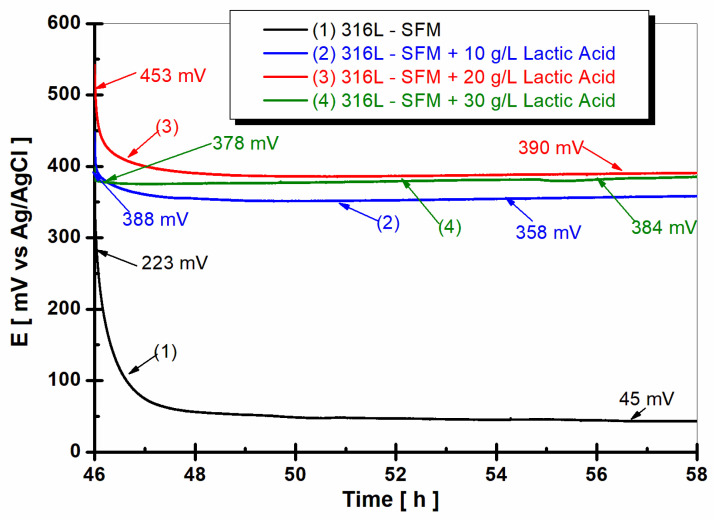
The evolution of the open circuit potential of 316L stainless steel was monitored for 12 h after 47 h from immersion in working solutions of (1) SFM; (2) SFM doped with 10 g·L^−1^ LA; (3) SFM doped with 20 g·L^−1^ LA; and (4) SFM doped with 30 g·L^−1^ LA.

After 47 h from immersion, the open circuit potential of 316L stainless steel immersed in the studied working solutions reveals a different behavior as shown in Figure 6. 

From Figure 6, it is observed that the open circuit potential of 316L stainless steel immersed in SFM shows a shift to a more positive value after 47 h of immersion. Thus, the value of the OCP starts from 223 mV vs. Ag/AgCl at the beginning of 12 h of monitoring, moving very slowly towards more negative values but becoming almost constant after two hours, at the end of the 12 h of monitoring having the value of 45 mV vs. Ag/Cl, as seen in the curve (1) from Figure 6.

For all concentrations of LA added to SFM, the values of the free potential of 316L stainless steel are at much more positive values compared to the value shown in the saliva solution. The values of the free potential are also very close for all lactic acid concentrations.

Doping the SFM with 10 gL^−1^ LA caused the free potential of the stainless steel to move towards more positive values compared with the value obtained after the first hour of immersion, having at the start of the 12 h of monitoring the value of 388 mV vs. Ag/AgCl and maintaining a flat line, reaching at the end of the 12 h of monitoring the value of 384 mV vs. Ag/AgCl, as seen in curve (2) from Figure 6.

The higher concentration of LA, 20 g·L^−1^, and 30 g·L^−1^ causes the further shift of OCP to a more positive value, having the values of 358 mV vs. Ag/AgCl, as seen in the curve (3), and 390 mV, respectively, as seen in the curve (4) from Figure 6. Regardless of the studied concentration, the presence of lactic acid in the saliva solution causes the free potential of the 316L stainless steel to move towards more positive values, as seen in Figure 6, curves (2), (3), and (4).

The OCP of 316L stainless steel, when immersed in SFM, has been observed to shift towards more positive values with the introduction of LA, as shown in Figure 6. It is well-documented that the OCP of a material represents the potential at which there is no net current flowing to or from the material and the electrolyte solution—essentially, the electrochemical equilibrium state [30].

In the case of 316L stainless steel, this equilibrium is largely influenced by the passive Cr_2_O_3_ film formed on its surface, which acts as a protective barrier against corrosion [24]. This film is the result of an inherent property of stainless steel, where chromium present in the alloy reacts with oxygen in the environment to form a thin, adherent, and protective oxide layer [31].

The observed shift in the OCP towards more positive values, upon the introduction of LA, could be interpreted as an enhancement in the passivation behavior of the stainless steel. Lactic acid may interact with the passive Cr_2_O_3_ film, potentially enhancing its protective properties and, thus, increasing the OCP [32]. However, this hypothesis warrants further experimental verification, as the specific electrochemical interactions between lactic acid and the passive Cr_2_O_3_ film on stainless steel remain to be elucidated in detail.

Nevertheless, the shift towards more positive OCP values with an increased lactic acid concentration suggests an increased resistance to corrosion, which could have significant implications for the longevity of stainless steel in biological applications, such as dental and orthodontic devices [33,34].

The interaction between lactic acid and the Cr_2_O_3_ film on the 316L stainless steel surface is proposed to enhance the film’s protective properties. The lactic acid may facilitate the formation or stabilization of the Cr_2_O_3_ film, leading to increased passivation and corrosion resistance at higher acid concentrations. This hypothesis is based on the observed shift in OCP values and is consistent with [30]. Further studies are needed to elucidate the exact mechanism of this interaction and its implications for the corrosion resistance of 316L stainless steel in lactic acid environments

### 3.4. EIS of 316L Steel after 59 h from Immersion (EIS_2_)

**Figure 7:**
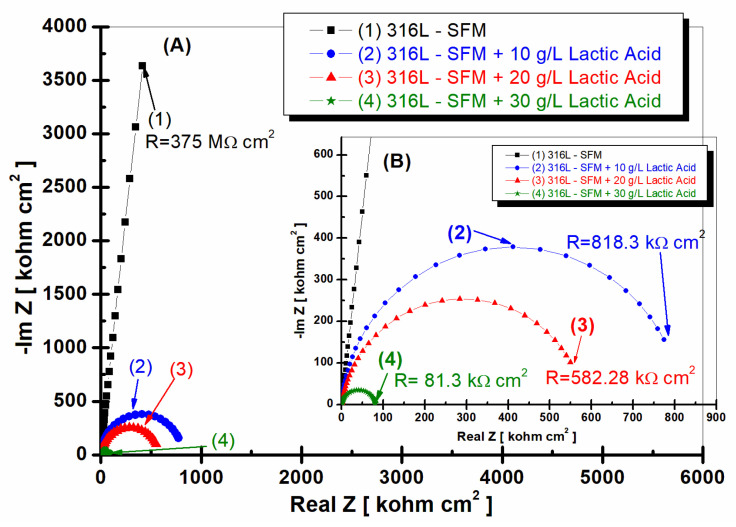
Nyquist plots of EIS results (filled symbols) and fitting with an equivalent electrical circuit (continuous line) of 316L stainless steel after 59 h from immersion in (1) SFM; (2) SFM doped with 10 gL^−1^ LA; (3) SFM doped with 20 gL^−1^ LA; and (4) SFM doped with 30 gL^−1^ LA. Layer (**A**)—all frequency range; layer (**B**)—zoom to high frequency to better see smaller EIS.

**Figure 8:**
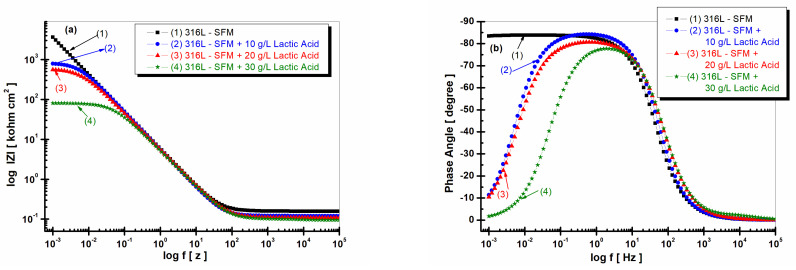
Bode plots of EIS results (filled symbols) and fitting with an equivalent electrical circuit (continuous line) of 316L stainless steel after 59 h from immersion in various solutions: (1) SFM; (2) SFM doped with 10 gL^−1^ LA; (3) SFM doped with 20 gL^−1^ LA; and (4) SFM was doped with 30 gL^−1^ LA. (**a**) Impedance modulus vs. log frequency (log f [Hz]); (**b**) phase angle vs. log frequency (log f [Hz]). The horizontal axis is on a logarithmic scale, with frequencies ranging from 10^−3^ to 10^−5^ Hz.

Figure 7 and Figure 8 show the EIS results performed on 316L stainless steel after 59 h of its immersion in SFM and saliva doped with different concentrations of LA, from 0 g·L^−1^ (saliva) to 10, 20, and 30 g·L^−1^.

From Figure 7, it is observed that, for all studied working solutions, the specific resistance of 316L stainless steel is decreasing.

For the 316L stainless steel immersed only in SFM, the specific resistance is found to be 375 MΩ·cm^2^, as seen in Figure 7, curve (1), which is higher compared with the resistance revealed for 316L stainless steel immersed in SFM doped with different concentrations of LA, as seen in Figure 7, curves (2), (3), and (4). The lowest resistance value is observed when 316L stainless steel is immersed in SFM doped with 30 g·L^−1^ LA, as seen in the curve (4) from Figure 7. 

Figure 8a,b shows the Bode plots of EIS results obtained after 59 h of immersion time. The impedance modulus of 316L stainless steel decreases with the increasing concentrations of LA added to the saliva solution, Figure 8a, curves (1), (2), (3), and (4). 

The lowest impedance modulus is revealed by 316L stainless steel immersed in Fusayama Meyer doped with 30 g·L^−1^ LA, as seen in Figure 8, curve (4). This behavior suggests that LA in a higher concentration affects the surface of 316L stainless steel, increasing its reactivity and susceptibility to degradation by the corrosion process.

The phase angle, Figure 8b, confirms the susceptibility to corrosion and increased reactivity of 316L stainless steel when LA is present in the saliva solution through its low values as seen in curves (2), (3), and (4) from Figure 8b. The phase angle of 316L stainless steel immersed in SFM shows a plateau at about 85 degrees over a wide range of frequencies, confirming good stability in saliva, as seen in Figure 8b, curve (1). When LA is present in saliva solution, the phase angle decreases rapidly after a maximum on a narrow range of frequencies, reaching values of approximately 10 degrees for 10 and 20 g·L^−1^ LA, as seen in Figure 8b, curves (2) and (3). The largest decrease in the phase angle is shown for 316L stainless steel immersed in saliva with 30 g·L^−1^ LA, as seen in Figure 8b, curve (4). This behavior once again confirms the instability of the 316L stainless steel surface in saliva doped with LA and its susceptibility to the corrosion degradation process.

### 3.5. Impedance Modulus at a Low Frequency of 0.01 Hz

**Figure 9:**
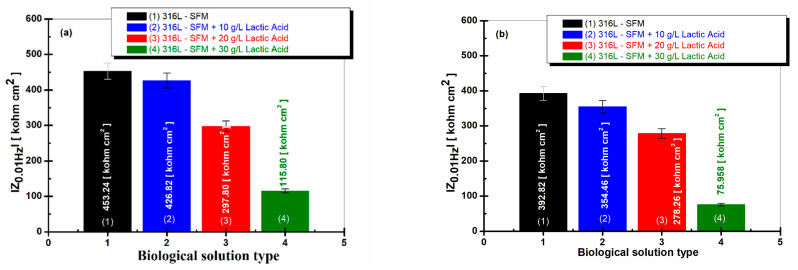
The low-frequency impedance modulus (at 0.01 Hz) for 316L stainless steel in SFM solution with different concentrations of LA: (**a**) after 1 h from immersion; and (**b**) after 59 h from immersion: (1) SFM; (2) SFM doped with 10 gL^−1^ LA; (3) SFM doped with 20 gL^−1^ LA; and (4) SFM was doped with 30 gL^−1^ LA.

The most suggestive comparative evaluation of the susceptibility of 316L stainless steel to degradation due to the corrosion process is given by the values of the impedance modulus obtained at the low frequency of 0.01 Hz, knowing that, at this low frequency, the corrosion processes that take place on the metal surfaces in corrosive environments can be evaluated [35,36,37]. For the studied working solutions, the values of the impedance modulus of the 316L steel are presented in Figure 9a after one hour of immersion and in Figure 9b after 59 h of immersion.

The differences between the low-frequency impedance modulus (at 0.01 Hz) for 316L stainless steel in SFM solution and saliva doped with different concentrations of LA confirm once more the increased reactivity of 316L stainless steel due to the lactic acid presence in saliva solution. 

The impedance modulus of 316L stainless steel immersed only in SFM shows a high value after one hour of immersion, respectively, 453.24 kohm·cm^2^, as seen in Figure 9a, column (1).

The value is kept in the same size range even after 59 h of immersion, respectively, 392.82 kohm·cm^2^, as seen in Figure 9b, column (1). 

The values of the 316L impedance modulus at 0.01 Hz immersed in SFM doped with different concentrations of LA are lower after one hour from immersion, as seen in Figure 9a, columns (2), (3), and (4), and decrease slowly after 59 h from immersion, as seen in Figure 9b, columns (2), (3), and (4). The lowest value is recorded at the concentration of 30 g·L^−1^ lactic acid in saliva, as seen in Figure 9a,b, column (4).

### 3.6. Linear Polarization and Tafel Plot Extrapolation

**Figure 10:**
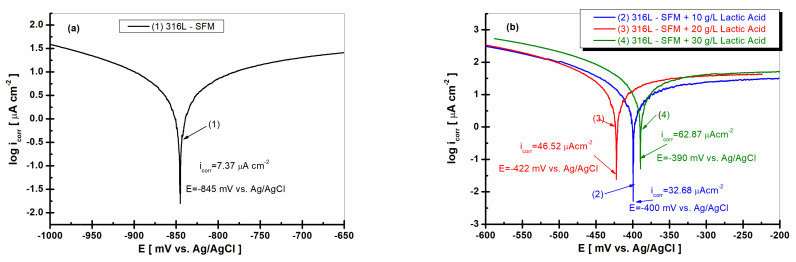
Corrosion rate as corrosion current density calculated from linear polarization by Tafel extrapolation plots: (1) SFM; (2) SFM doped with 10 gL^−1^ LA; (3) SFM doped with 20 gL^−1^ LA; and (4) SFM was doped with 30 gL^−1^ LA.

In Figure 10a,b, the corrosion current densities of 316L stainless steel are shown. As can be seen, the corrosion potential of 316L stainless steel is shifted to more positive values, shown in Figure 10b, compared with the corrosion potential of 316L immersed in SFM, which revealed a more negative value, shown in Figure 10a.

The corrosion current densities confirm the behavior of 316L stainless steel immersed in the SFM solution and in saliva doped with different concentrations of LA. Thus, the corrosion current density of the 316L steel immersed in saliva registers the lowest value, respectively, 7.37 μA·cm^−2^, as seen in Figure 10a, curve (1).

In Figure 10, the Tafel plots for 316L stainless steel under various conditions are presented. The region showcasing minimal change in current density is indicative of the passivation plateau. This plateau is a manifestation of the stability of the chromium oxide passive film formed on the surface of 316L stainless steel. The end of this plateau, where a noticeable increase in current density is observed, denotes the pitting potential, a critical parameter that evaluates the susceptibility of the material to pitting corrosion.

The observed passivation plateau and the derived pitting potential values are consistent with the literature findings. Specifically, the pitting potential values obtained in our study align with those reported for 316L stainless steel in similar environments [38].

The significance of the Tafel behavior, especially the passivation plateau, lies in its ability to provide insights into the corrosion resistance of 316L stainless steel. The stability and duration of this plateau are indicative of the robustness of the passive film, with a longer plateau suggesting enhanced corrosion resistance. Conversely, the onset of the pitting potential, as derived from the Tafel plots, provides a threshold beyond which the material becomes susceptible to localized pitting corrosion.

Corrosion current values for 316L stainless steel immersed in saliva doped with LA working solutions increase proportionally with the decrease in specific resistance resulting from EIS data, as seen in Figure 10b, curves (2), (3), and (4). 

Thus, the lowest corrosion current density in the presence of LA in the saliva is 32.68 μA·cm^−2^ for 10 g·L^−1^ LA. The value increases to 46.52 μA·cm^−2^ for the concentration of 20 gL^−1^ LA and reaches the highest value of 62.87 μA·cm^−2^ for the concentration of 30 g·L^−1^ LA.

This section aims to elucidate the observed corrosion behavior of 316L stainless steel when exposed to the tested solutions, with a particular emphasis on the notable absence of pits. The absence of pits in the solution can be attributed to the enhanced protective properties of the passive film formed on the 316L stainless steel surface under specific experimental conditions. Such behavior aligns with findings from Luo, H. et al., where the stability of the passive film effectively prevented pitting corrosion under analogous conditions [39].

The significance of this observation is paramount. The absence of pitting corrosion suggests that the 316L stainless steel, under the conditions tested, exhibits superior corrosion resistance. This has profound implications for its potential applications, especially in environments where such corrosion behavior is detrimental.

The Tafel behavior observed for 316L stainless steel, characterized by a distinct passivation plateau and a derived pitting potential, underscores its corrosion-resistant properties. The findings from this study, supported by literature comparisons, offer a comprehensive understanding of the factors influencing the corrosion behavior of 316L stainless steel.

### 3.7. Optical Microscopy Analysis before and after Corrosion

**Figure 11:**
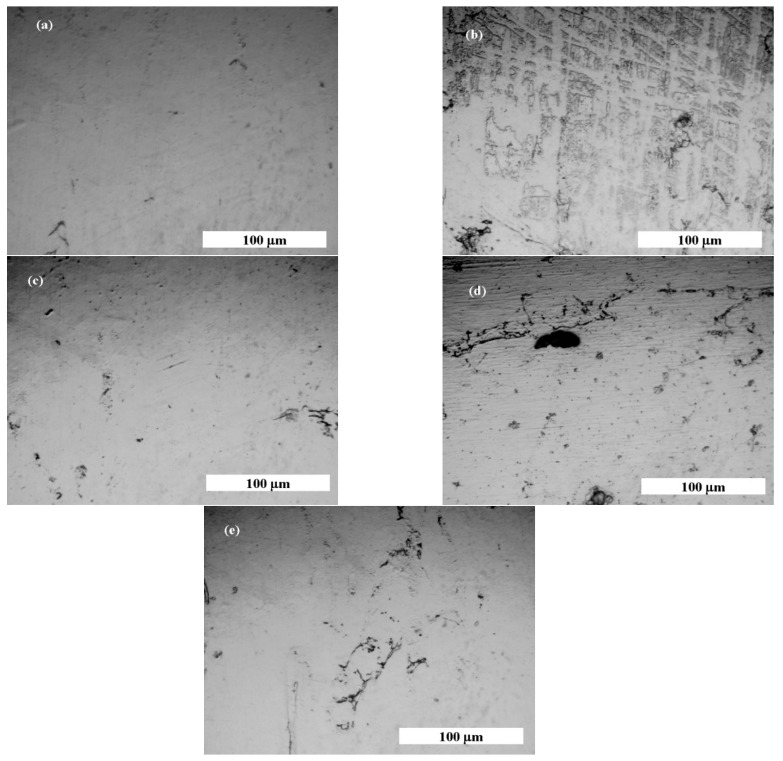
Optical microscopy micrographs of 316L stainless steel: (**a**) before corrosion tests; (**b**) after immersion in SFM; (**c**) after immersion in SFM doped with 10 g·L^−1^ LA; (**d**) after immersion in SFM doped with 20 g·L^−1^ LA; and (**e**) after immersion in SFM doped with 30 gL^−1^ LA.

The micrographs shown in Figure 11 obtained by optical microscopy complete the electrochemical results regarding the evaluation of the corrosion behavior of the 316L stainless steel immersed in the Fusayama Meyer saliva solution and saliva doped with lactic acid.

Figure 11 presents optical microscopy images of 316L stainless steel. The untouched surface of the steel, devoid of scratches or imperfections, is illustrated in Figure 11a. After being immersed in SFM, Figure 11b reveals initial indications of localized corrosion. Immersion in saliva containing 10 gL^−1^ LA shows the surface mainly undergoing generalized corrosion, as depicted in Figure 11c. In contrast, images from immersion in saliva with concentrations of 20 and 30 gL^−1^ LA, seen in Figure 11d,e, display a mix of both localized and generalized corrosion. We have added arrows to the figure to help readers differentiate between generalized and localized corrosion areas.

In these images, generalized corrosion manifests as a consistent thinning across the material, while localized corrosion appears as pits, crevices, or other concentrated damage on the surface. These distinctions are drawn from the visual assessment of corrosion patterns on the 316L stainless steel after its immersion in different solutions.

Our study’s insights into the corrosion behavior of 316L stainless steel have both parallels and differences when compared to existing literature. For instance, our observations align with Liu et al. (2023) in that the passive film is vital for corrosion protection [37]. Yet, the specific attributes and actions of the passive film on 316L stainless steel vary considerably from those seen on 3D-printed NiTi shape memory alloys.

While our research offers a significant understanding of the corrosion behavior of 316L stainless steel in lactic acid settings via electrochemical tests, we recognize the need for more in-depth passive film characterization, possibly through XPS or AES tests. Future studies will include these tests to further explore the composition and protective qualities of passive films on 316L stainless steel in lactic acid conditions.

## 4. Conclusions

This study evaluated the corrosion behavior of 316L stainless steel in a saliva fluid medium (SFM) with varying concentrations of lactic acid (LA), aiming for applications in orthodontics. The results indicated a positive shift in open circuit potential (OCP) values and an increase in the corrosion rate, measured as the corrosion current density, with higher LA concentrations in saliva solutions. Electrochemical impedance spectroscopy (EIS) revealed a significant decrease in specific resistance after one hour in all LA-doped saliva solutions, suggesting the reduced corrosion resistance of 316L stainless steel under these conditions. This trend persisted over extended immersion periods. Optical microscopy analysis corroborated the electrochemical findings, showing surface changes on the steel before and after corrosion tests.

The study underscores the need for careful material selection and potential surface treatments to enhance the corrosion resistance and durability of orthodontic appliances in environments containing lactic acid.

## Figures and Tables

**Table 1:** Chemical composition of 316L stainless steel.

Element and Percent Concentration						
C	Mn	Si	Mo	Ni	Cr	Fe
[%]	[%]	[%]	[%]	[%]	[%]	[%]
≥0.03	≥2	1	2.0–2.5	11–14	16.5–18.5	Balance

**Table 2:** Composition of biological body fluid Saliva Fusayama Meyer.

Nr. crt.	Chemical Compound	Concentration g/L
1	Potassium chloride (KCl)	0.4
2	Sodium chloride (NaCl)	0.4
3	Calcium chloride dihydrate (CaCl_2_·2H_2_O)	0.906
4	Monosodium phosphate dihydrate (Na_2_HPO_4_·H_2_O)	0.69
5	Sodium sulfide nonahydrate (Na_2_S·9H_2_O)	0.005
7	Urea (C_6_H_12_O_6_)	1.0

**Table 3:** The physicochemical characteristics of biological SFM solution and modified SFM solution with LA.

Nr. crt.	Solution Type	pH	Conductivity [mS/cm]	Salinity [ppt]
1	SFM	5.80 ± 0.3	2.73 ± 0.1	1.4 ± 0.1
2	SFM + 10 g/L LA	2.30 ± 0.2	4.24 ± 0.2	2.3 ± 0.1
3	SFM + 20 g/L LA	2.15 ± 0.3	4.79 ± 0.1	2.8 ± 0.1
4	Hank + 30 g/L LA	1.99 ± 0.2	5.20 ± 0.1	2.9 ± 0.1

## Data Availability

All data generated or analyzed during this study are included in this published article.

## References

[1] Poursaee A. (2023). Corrosion of Steel in Concrete Structures.

[2] Badd N.R. (2008). Stainless steel in construction: A review of research, applications, challenges and opportunities. J. Constr. Steel Res..

[3] Marcus P., Maurice V. (2017). Atomic level characterization in corrosion studies. Philos. Trans. R. Soc. A Math. Phys. Eng. Sci..

[4] Harsimran S., Santosh K., Rakesh K. (2021). Overview of corrosion and its control: A critical review. Proc. Eng. Sci..

[5] Sanni O., Iwarere S.A., Daramola M.O. (2023). Introduction: Corrosion basics and corrosion testing. Electrochemical and Analytical Techniques for Sustainable Corrosion Monitoring.

[6] Nosei L., Farina S., Ávalos M., Náchez L., Gómez B., Feugeas J. (2008). Corrosion behavior of ion nitrided AISI 316L stainless steel. Thin Solid Films.

[7] Dewidar M.M., Khalil K.A., Lim J.K. (2007). Processing and mechanical properties of porous 316L stainless steel for biomedical applications. Trans. Nonferrous Met. Soc. China.

[8] Cigada A., Rondelli G., Vicentini B., Giacomazzi M., Roos A. (1989). Duplex stainless steels for osteosynthesis devices. J. Biomed. Mater. Res..

[9] Wang Q., Zhang M., Yang C., Yang Y., Zhou E., Liu P., Jin D., Xu D., Wu L., Wang F. (2022). Oral microbiota accelerates corrosion of 316L stainless steel for orthodontic applications. J. Mater. Sci. Technol..

[10] Oh K.-T., Choo S.-U., Kim K.-M., Kim K.-N. (2005). A stainless steel bracket for orthodontic application. Eur. J. Orthod..

[11] Oh K.-T., Kim Y.-S., Park Y.-S., Kim K.-N. (2004). Properties of super stainless steels for orthodontic applications. J. Biomed. Mater. Res..

[12] Walczak J., Shahgaldi F., Heatley F. (1998). In vivo corrosion of 316L stainless-steel hip implants: Morphology and elemental compositions of corrosion products. Biomaterials.

[13] Kocijan A., Merl D.K., Jenko M. (2011). The corrosion behaviour of austenitic and duplex stainless steels in artificial saliva with the addition of fluoride. Corros. Sci..

[14] Threlfall A.G., Pilkington L., Milsom K.M., Blinkhorn A.S., Tickle M. (2005). General dental practitioners’ views on the use of stainless steel crowns to restore primary molars. Br. Dent. J..

[15] Dumitriu H.T. (2015). Tratat de Parodontologie.

[16] Caufield P.W., Schön C.N., Saraithong P., Li Y., Argimón S. (2015). Oral Lactobacilli and Dental Caries: A Model for Niche Adaptation in Humans. J. Dent. Res..

[17] Castillo Martinez F.A., Balciunas E.M., Salgado J.M., Domínguez González J.M., Converti A., Oliveira R.P.D.S. (2013). Lactic acid properties, applications and production: A review. Trends Food Sci. Technol..

[18] Lemos J.A., Palmer S.R., Zeng L., Wen Z.T., Kajfasz J.K., Freires I.A., Abranches J., Brady L.J. (2019). The Biology of Streptococcus mutans. Microbiol. Spectr..

[19] Arroyo B., Andrea L., Álvarez J.A., Cicero S., Lacalle R. (2020). Analysis of Samples Cleaning Methods Prior to Hydrogen Content Determination in Steel. Metals.

[20] Revilla R.I., Wouters B., Andreatta F., Lanzutti A., Fedrizzi L., De Graeve I. (2020). EIS comparative study and critical Equivalent Electrical Circuit (EEC) analysis of the native oxide layer of additive manufactured and wrought 316L stainless steel. Corros. Sci..

[21] Zaheer K. (2001). Formation of chromium (V)-lactic acid complex in the chromic acid oxidation of lactic acid. Indian J. Chem. Sect. A Inorg. Phys. Theor. Anal. Chem..

[22] Zhang Y., Luo H., Zhong Q., Yu H., Lv J. (2019). Characterization of Passive Films Formed on As-received and Sensitized AISI 304 Stainless Steel. Chin. J. Mech. Eng..

[23] Parangusan H., Bhadra J., Al-Thani N. (2021). A review of passivity breakdown on metal surfaces: Influence of chloride- and sulfide-ion concentrations, temperature, and pH. Emergent Mater..

[24] Wang J., Wang J., Ming H., Zhang Z., Han E. (2018). Effect of pH on corrosion behavior of 316L stainless steel in hydrogenated high temperature water. Mater. Corros..

[25] Li D., Wang J., Chen D., Liang P. (2014). Influences of pH value, temperature, chloride ions and sulfide ions on the corrosion behaviors of 316L stainless steel in the simulated cathodic environment of proton exchange membrane fuel cell. J. Power Sources.

[26] Córdoba-Torres P., Mesquita T.J., Devos O., Tribollet B., Roche V., Nogueira R.P. (2012). On the intrinsic coupling between constant-phase element parameters α and Q in electrochemical impedance spectroscopy. Electrochim. Acta.

[27] Jorcin J.-B., Orazem M.E., Pébère N., Tribollet B. (2006). CPE analysis by local electrochemical impedance spectroscopy. Electrochim. Acta.

[28] Gharbi O., Tran M.T., Tribollet B., Turmine M., Vivier V. (2020). Revisiting cyclic voltammetry and electrochemical impedance spectroscopy analysis for capacitance measurements. Electrochim. Acta.

[29] Bard A.J., Faulkner L.R. (2000). Electrochemical Methods: Fundamentals and Applications.

[30] Oliveira N.T.C., Guastaldi A.C. (2009). Electrochemical stability, and corrosion resistance of Ti–Mo alloys for biomedical applications. Acta Biomater..

[31] Marcus P. (2011). Corrosion Mechanisms in Theory and Practice.

[32] O’Laoire C., Timmins B., Kremer L., Holmes J.D., Morris M.A. (2006). Analysis of the Acid Passivation of Stainless Steel. Anal. Lett..

[33] Mabilleau G., Bourdon S., Joly-Guillou M., Filmon R., Baslé M., Chappard D. (2006). Influence of fluoride, hydrogen peroxide and lactic acid on the corrosion resistance of commercially pure titanium. Acta Biomater..

[34] Manivasagam G., Dhinasekaran D., Rajamanickam A. (2010). Biomedical Implants: Corrosion and its Prevention—A Review. Recent Patents Corros. Sci..

[35] Matsumura M., Oka Y., Hiura H., Yano M. (1991). The role of passivating film in preventing slurry erosion-corrosion of austenitic stainless steel. ISIJ Int..

[36] Riđošić M., Bučko M., Salicio-Paz A., García-Lecina E., Živković L.S., Bajat J.B. (2021). Ceria Particles as Efficient Dopant in the Electrodeposition of Zn-Co-CeO_2_ Composite Coatings with Enhanced Corrosion Resistance: The Effect of Current Density and Particle Concentration. Molecules.

[37] Varmaziar S., Atapour M., Hedberg Y.S. (2022). Corrosion and metal release characterization of stainless steel 316L weld zones in whey protein solution. npj Mater. Degrad..

[38] Voisin T., Shi R., Zhu Y., Qi Z., Wu M., Sen-Britain S., Zhang Y., Qiu S.R., Wang Y.M., Thomas S. (2022). Pitting Corrosion in 316L Stainless Steel Fabricated by Laser Powder Bed Fusion Additive Manufacturing: A Review and Perspective. JOM.

[39] Luo H., Su H., Dong C., Li X. (2017). Passivation and electrochemical behavior of 316L stainless steel in chlorinated simulated concrete pore solution. Appl. Surf. Sci..

